# Mechanisms of HDACs in cancer development

**DOI:** 10.3389/fimmu.2025.1529239

**Published:** 2025-04-07

**Authors:** Ying Zhang, Haotian Wang, Zhumei Zhan, Lin Gan, Out Bai

**Affiliations:** ^1^ Department of Hematology, The First Hospital of Jilin University, Changchun, China; ^2^ Department of Hematology, Shandong Provincial Hospital Affiliated to Shandong First Medical University, Jinan, Shandong, China; ^3^ Department of Neurology and Neuroscience Center, The First Hospital of Jilin University, Changchun, China

**Keywords:** histone acetyltransferases, histone deacetylases, cancer, HDACi, lymphoma

## Abstract

Histone deacetylases (HDACs) are a class of epigenetic regulators that play pivotal roles in key biological processes such as cell proliferation, differentiation, metabolism, and immune regulation. Based on this, HDAC inhibitors (HDACis), as novel epigenetic-targeted therapeutic agents, have demonstrated significant antitumor potential by inducing cell cycle arrest, activating apoptosis, and modulating the immune microenvironment. Current research is focused on developing highly selective HDAC isoform inhibitors and combination therapy strategies tailored to molecular subtypes, aiming to overcome off-target effects and resistance issues associated with traditional broad-spectrum inhibitors. This review systematically elaborates on the multidimensional regulatory networks of HDACs in tumor malignancy and assesses the clinical translation progress of next-generation HDACis and their prospects in precision medicine, providing a theoretical framework and strategic reference for the development of epigenetic-targeted antitumor drugs.

## 1 Introduction

In eukaryotic cells, DNA is packaged into chromatin, with the nucleosome serving as its fundamental unit. Each nucleosome consists of approximately 148 base pairs of DNA wrapped around an octamer of core histone proteins, comprising two copies each of H2A, H2B, H3, and H4 (1). This packaging typically creates a repressive environment for gene expression. Therefore, transcriptional activation often requires chromatin modifications. Histone deacetylases (HDACs) and histone acetyltransferases (HATs) are two enzymes that regulate chromatin structure and function by adding and removing acetyl groups on lysine residues of core nucleosomal histones, respectively. The acetylation of histones H3 and H4 neutralizes the positive charge of lysine residues, leading to chromatin relaxation and enhanced accessibility for transcriptional activation. In contrast, histone deacetylation promotes chromatin condensation, thereby suppressing gene transcription (2, 3). Consequently, the dynamic equilibrium between acetylation and deacetylation levels plays a pivotal role in regulating physiological processes, cellular fate determination, and the pathogenesis of diseases. Growing evidence suggests that members of the HDAC family exhibit extensive functional heterogeneity during tumorigenesis and cancer progression. These enzymes contribute to oncogenic activation, inactivation of tumor suppressor pathways, metabolic adaptation, and alterations in the immune microenvironment, collectively driving malignant progression (4). Given the tumor-promoting properties of HDACs, targeted inhibition of HDAC activity has emerged as a critical therapeutic strategy in oncology. Although first-generation pan-HDAC inhibitors (HDACis), such as Vorinostat and Romidepsin, have been clinically approved for treating specific lymphomas, their off-target effects and drug resistance issues have limited broader clinical applications (5). Current research focuses on developing subtype-selective HDACis, exploring biomarker-guided precision dosing regimens, and designing combination therapeutic strategies integrating HDACis with chemotherapy, immune checkpoint inhibitors, or targeted agents to overcome current therapeutic efficacy limitations. This review systematically elucidates the pathological regulatory networks of HDACs in malignancies, dissects the mechanisms of action and pharmacological properties of HDACis, summarizes advancements in preclinical and clinical studies, and discusses future directions to optimize epigenetic-targeted therapies, thereby providing a scientific foundation for refining antitumor strategies.

## 2 Histone deacetylases

**Figure 1:**
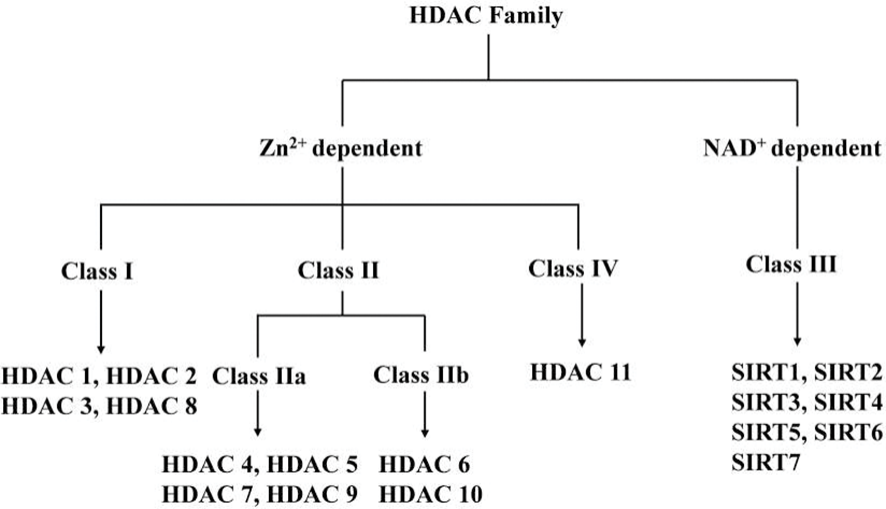
HDAC Family.

The enzymatic capacity to remove acetyl groups from histones was first documented in 1969 through biochemical characterization of calf thymus extracts (6), though initial purification efforts using conventional chromatographic techniques proved unsuccessful. The field underwent transformative advancement in 1996 with the molecular cloning of HDAC1, the first bona fide histone deacetylase identified (7). Subsequent genomic analyses revealed 18 human HDACs categorized into four distinct classes based on sequence homology, subcellular localization, and enzymatic cofactor requirements. Notably, Classes I, II, and IV enzymes (HDAC1-11) are zinc-dependent hydrolases, whereas Class III sirtuins (SIRT1-7) utilize NAD^+^ as an essential cofactor for their catalytic activity (**Figure 1**). This phylogenetic classification reflects both conserved catalytic domains and divergent regulatory mechanisms across evolutionary lineages.

### 2.1 Class I (HDAC1, HDAC2, HDAC3, HDAC8)

The Class I HDACs (HDAC1, 2, 3 and 8) are most closely related to the yeast (*Saccharomyces cerevisiae*) transcriptional regulator Rpd3 (8). It has now been widely stated in the literature that Class I HDACs are located in the nucleus and are widely expressed. More thorough findings reveal that HDAC3 expression is restricted to certain tissues, and HDAC1, HDAC2, HDAC3, and HDAC8 can localize in the cytoplasm or specialized cellular organelles (9).

### 2.2 Class II (HDAC4, HDAC5, HDAC6, HDAC7, HDAC9, HDAC10)

The Class II HDACs have homology to yeast HDAC1, and are further subdivided into two subclasses, IIa and IIb, based on sequence homology and domain organization. The Class IIa HDACs, HDAC4, -5, -7, and -9, contain a highly conserved C-terminal deacetylase catalytic domain (*420 amino acids) homologous to yHda1 and share an N-terminal domain (*450–600 amino acids) with no similarity to HDACs in other classes. The N-terminal domain mediates interactions with the myocyte enhancer factor 2 transcription factor family, transcriptional corepressor C-terminal binding protein, and others. Class IIa HDACs shuttle between the cytoplasm and nucleus and their expression is tissue-specific. The Class IIb HDACs, HDAC6 and -10 are characterized by the presence of two catalytic HDAC domains arranged in tandem. Class IIb HDACs are predominantly cytoplasmic and expressed in a limited number of cell types (10, 11).

### 2.3 Class III (SIRT1, SIRT2, SIRT3, SIRT4, SIRT5, SIRT6, and SIRT7)

Class III HDACs are distinct from Class I and II and are homologous of the yeast silent information regulator 2 (Sir2). Sir2 enzymes (or sirtuins) are NAD (+)-dependent deacetylases that regulate gene silencing, aging, and energy metabolism. Earlier experiments showed that overexpression of Sir2 in yeast induced global deacetylation of histones, suggesting that Sir2 is an HDAC (12). Later, in a study by Frye et al. (13) the bacterial homolog of Sir2, *cobB*, was found to have ribosyltransferase activity, leading to experiments showing that Sir2 can also transfer adenosine-ribose (ADP-ribose) from nicotinamide adenine nucleotide (NAD). Subsequently, it was confirmed that Sir2 is an NAD-dependent HDAC and that the ADP-ribosylation of acetylated lysine residues is an intermediate state of the Sir2-catalyzed enzymatic reaction (14). Only Class III enzymes uses NAD as a cofactor. Therefore, they are referred to as NAD-dependent HDACs. Each of the seven mammalian sirtuin proteins (called SIRT1-SIRT7) has a different subcellular localization. SIRT1, SIRT6 and SIRT7 are localized in the nucleus, while SIRT2 is mainly cellular membrane and SIRT3, SIRT4 and SIRT5 seem to be present only in mitochondria. Although much is known about SIRT1, relatively little is known about the other Sirt family proteins (15). What is interesting is that some sirtuins have been recently shown to have additional enzymatic activities in addition to deacetylation, such as SIRT3 showing additional norylase activity, SIRT4 showing ADP-ribosyltransferase activity, SIRT5 with desmalonylase, desuccinylase and deglutaminase activities, and SIRT6 showing deacetylase and demyristoylase activities, which were further elaborated in the study by Chen et al (16).

It cannot be ignored that the role of some current sirtuins in tumorigenesis is still controversial. In a study by Heltweg et al. (17) it was demonstrated that SIRT1 is expressed at higher levels in cancer cells and promotes tumorigenesis through deacetylation of lysine 382 in Burkitt’s lymphoma cells. However, in a mouse model of cancer constructed by Firestein et al. (18) increased SIRT1 expression inhibited cell proliferation and tumor formation.

### 2.4 Class IV (HDAC11)

HDAC11 was first reported in 2002 only and is the smallest of the HDAC isoforms, with its catalytic domain accounting for > 80% of the protein sequence. It is mainly localized in the nucleus and plays an important role in immune regulation as a transcriptional regulator (19). HDAC11 is predominantly expressed in smooth muscle, heart, kidney and brain tissues (20), and is likely to be preferentially expressed in the gallbladder (21).

The location and biological functions of HDACs are detailed in **Table 1**.

**Table 1:** Location and biological function of HDACs.

Class	Type	Location	Chromosomal Location	Tissue Expression	Biological Function
ClassI	HDAC 1	Nucleus	1p35.2-p35.1	Ubiquitous	Promote cell proliferation, inhibit apoptosis;
	HDAC 2		6q21		
	HDAC 3		5q31.3		
	HDAC 8		Xq13.1		
ClassIIa	HDAC 4	Nucleus Cytoplasm	2q37.3	Heart, smooth muscle, brain	Promote angiogenesis;
	HDAC 5		17q21.31		
	HDAC 7		12q13.11	Heart, placenta, Pancreas, smooth muscle	
	HDAC 9		7p21.1	Smooth muscle, brain	
ClassIIb	HDAC 6	Cytoplasm	Xp11.23	Kidney, liver, heart, pancreas	Promote angiogenesis, cell migration;
	HDAC 10		2q13.33	Spleen, kidney, liver	
Class III	SIRT 1	Nucleus Cytoplasm	10q21.3	Ubiquitous	Apoptosis, autophagy, cell proliferation
	SIRT 2	Nucleus	19q13.2		
	SIRT 3	Mitochondria	11p15.5		
	SIRT 4		12q24.31		
	SIRT 5		6p23		
	SIRT 6	Nucleus	19p13.3		
	SIRT 7		17q25.3		
ClassIV	HDAC11	Nucleus	3p25.1	Heart, smooth muscle, brain	Inhibit cell migration

## 3 Histone deacetylases inhibitors

There are several main classes of HDAC inhibitors (HDACis), including those based on hydroxamic acid (e.g., suberoylanilide hydroxamic (SAHA), pyroxamide, trichostatin A (TSA), oxamflatin, cyclic hydroxamic acid-containing peptides (CHAP)s; LAQ824; BL1521); cyclic tetra/peptide (e.g., depsipeptide, trapoxin, apicidin, CHAPs); synthetic benzamide derivatives (e.g., MS-275 and CI-994); cyclic tetrapeptides (e.g., depsipeptide, trapoxin, apicidin, CHAPs); and short-chain fatty acids (e.g., sodium butyrate (SB), AN-9; phenylbutyrate (PB);phenylacetate (PA); valproic acid) (22).

The recent discovery of hydrazide-based HDACi further increases the diversity of HDACi (23, 24). Four HDACi are currently approved by the FDA (25–29) (**Table 2**). The first class of HDACi is hydroxyamide acid-based HDACi. Vorinostat is well-known as the first marketed HDACi with nanomolar affinity for HDACs. Initially, relevant studies suggested that this compound was able to inhibit all HDACs. However, further testing demonstrated that it could only inhibit HDAC 1, 2, 3, and 6 at reasonable concentrations (30). Currently, vorinostat is FDA-approved for the treatment of cutaneous T-cell lymphoma (CTCL). Amino-benzamide-based HDACi are the first inhibitors to selectively target Class I HDACs (31), and enzyme kinetic studies have shown that the aminobenzamide motif has a tight binding mechanism (slow-on/slow-off) and differs from classical fast on/fast off kinetics associated with hydroxamide acid-based HDACi (32–34). Entinostat is the first amino-benzamide-based HDACi to reach clinical trials (35). Recently, Wang et al (24) reported a novel HDACi family with a previously unutilized motif in HDACi.

**Table 2:** HDACis currently under clinical investigations.

HDACis	Specificity	Tumor Type	Clinical Trail	References
Hydroxamic acid				
Vorinostat	ClassI, II and IV	CTCL	FDA approved in 2006	(148)
Belinostat	ClassI, II and IV	PTCL	FDA approved in 2014	(149)
Panobinostat	Class I, II and IV	MM	FDA approved in 2015	(150)
Resminostat	Class I and II	Colorectal, HCC, HL	Phase II trial	(151)
Givinostat	Class I and II	CLL, HL, MM	Phase II trial	(152)
Pracinostat	Class I,II, and IV	MDS, AML	Phase II trial	(153–155)
Abexinostat	Class I and II	CLL, HL, Non-HL, Solid tumors	Phase I trial	(156–158)
Quisinostat	Class I and II	MM, advanced solid tumor, CTCL	Phase Ib/I/II trail	(159–161)
CUDC-101	Class I and II	Squamous Cell Carcinoma, advanced solid tumors	Phase I trial	(162, 163)
CUDC-907	Class I and II	Lymphoma, MM,	Phase I/II trial	(164–166)
CHR 3996	Class I	Solid tumors	Phase I trail	(167)
MPT0E028	HDAC1, 2, 6	Solid tumor, B-cell lymphoma	Phase I trial	(168)
Cyclic peptides				
Romidepsin	Class I	CTCL, PTCL	FDA approved in 2009 and 2011	(26, 27)
Benzamides				
Entinostat	Class I	Solid tumors, AML, ABL	Phase I/II/III trial	(168–187)
Chidamide	HDAC 1,2,3,10	PTCL, AITL, ENKTCL, lymphoma, Breast cancer, DLBCL	Phase Ib/II/III trial	(188–194)
Ricolinostat	HDAC 6	MM, Lymphoma	Phase Ib/II trial	(195, 196)
Tacedinaline	Class I	Advanced pancreatic cancer, MM	Phase II/III trial	(151, 197)
Mocetinostat	Class I and IV	cHL, R/R lymphoma,	Phase II trial	(198, 199)
Fatty acids				
Valproic acid	Class I and II	Solid and hematological tumors	Phase I/II trial	(200)
Phenylbutyrate	Class I and II	Solid and hematological tumors	Phase I/II trial	(200)
AR-42	Class I and IIb	AML, Solid tumor, lymphoma	Phase I/II trial	(158, 201, 202)
Pivanex	Classes I and II	NSCLC, Myeloma, CLL	Phase II trial	(167, 203)

CTCL, cutaneous T-cell lymphoma; PTCL, peripheral T-cell lymphoma; MM, multiple myeloma; MDS, myelodysplastic syndromes; AML, acute myeloid leukemia; ABL, acute biphenotypic leukemia; DLBCL, diffuse large B-cell lymphoma; NSCLC, non-small cell lung cancer; AITL, angioimmunoblastic T-cell lymphoma; ENKTCL, extranodal natural killer/T-cell lymphoma; cHL, classical Hodgkin lymphoma; R/R lymphoma, relapsed or refractory lymphoma.

## 4 Mechanisms of HDACs in cancer development

**Figure 2:**
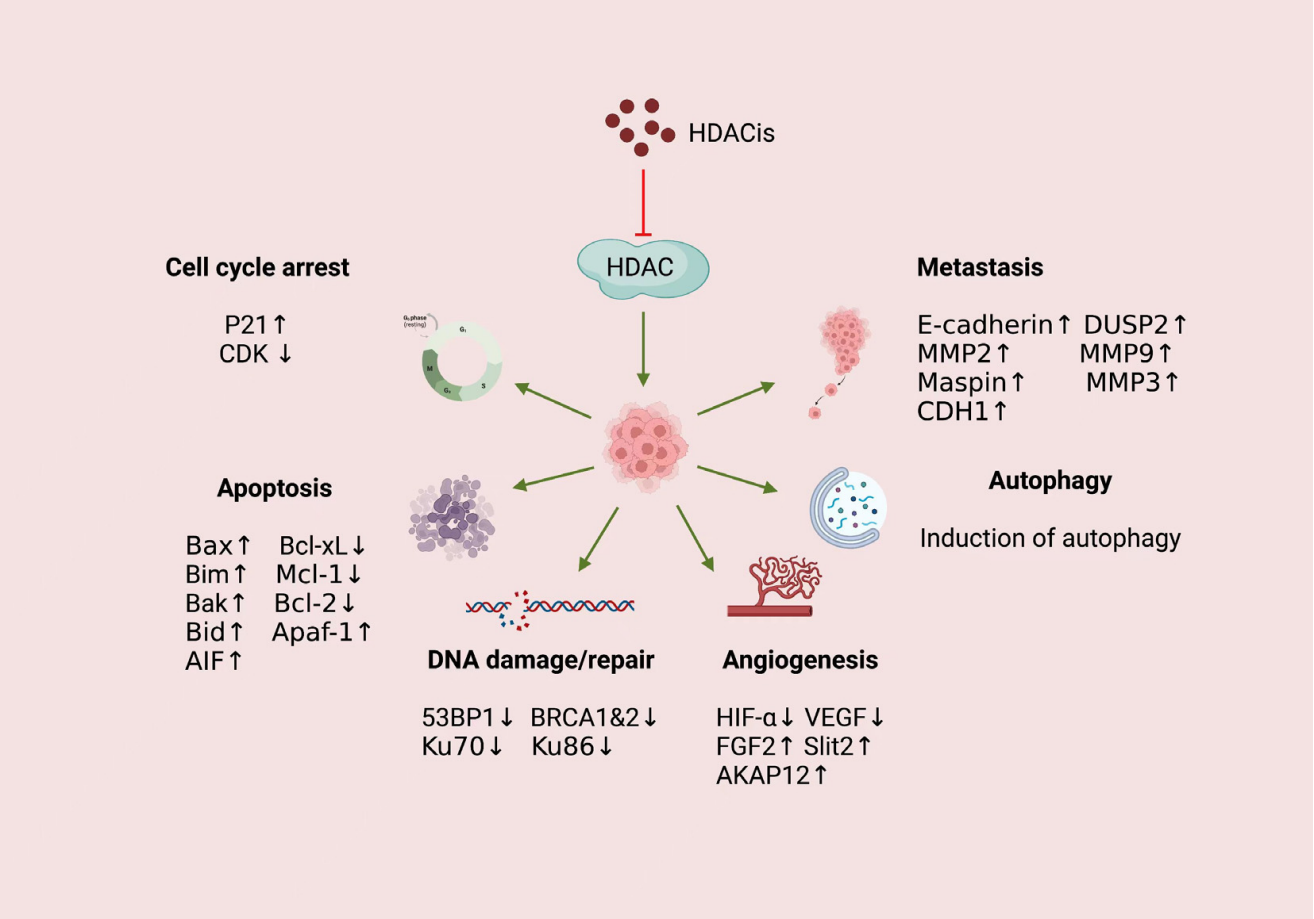
Mechanism of action of HDACis in cancer.

While the substrate specificity and biological functions of individual HDAC isoforms remain incompletely characterized, accumulating evidence has unequivocally established HDACs as central epigenetic orchestrators of oncogenic processes. Through dynamic deacetylation of both histone (e.g., H3K9, H4K16) and non-histone substrates (e.g., p53, STAT3, HIF-1α), HDACs mechanistically govern six hallmark cancer pathways: (1) cell cycle progression via CDK inhibitor silencing, (2) apoptosis resistance through Bcl-2 family modulation, (3) DNA damage tolerance by repair factor inactivation, (4) autophagic flux dysregulation, (5), VEGF-driven angiogenesis, and (6) EMT-mediated metastatic dissemination. In particular, recent studies have given great attention to the epigenetic regulation involved in HDAC. This functional pleiotropy positions HDACs as master regulators linking epigenetic plasticity to tumor microenvironment remodeling, but precise mechanistic mapping requires systematic subtype and context-specific interrogation (**Figure 2**).

### 4.1 Cell cycle

The precise coordination of cell cycle progression is fundamental to maintaining genomic stability and cellular homeostasis, governed by a tightly regulated network of cyclin-dependent kinases (CDKs), checkpoint proteins, and epigenetic modifiers. Among these regulators, histone deacetylases (HDACs) have emerged as critical players that bridge epigenetic dynamics with cell cycle control. By modulating the acetylation status of histones (e.g., H3K9, H4K16) and non-histone substrates (e.g., p53, E2F, Rb), HDACs exert spatiotemporal control over the transcription of cell cycle-related genes, DNA replication licensing, and mitotic fidelity.

#### 4.1.1 G_1_/S transition

The G_1_/S phase transition represents a critical checkpoint in the cell cycle, determining whether a cell commits to proliferation or exits the cycle into quiescence. Previous studies have confirmed that HDAC1 and HDAC2 bind to the promoter regions of the *p21* and *p57* genes, thereby inhibiting their expression and regulating the transition from the G_1_ phase to the S phase of the cell cycle (5). Fan et al. found that knockdown of HDAC5 led to a significant upregulation of *p21* and a notable downregulation of cyclin D1 and CDK2/4/6, resulting in a strong G_1_-phase cell cycle arrest and inhibiting cell proliferation (36). Similarly, Qiu et al. (37) demonstrated that HDACi induce G_1_ arrest by upregulating *p21*. HDACi also reduce CDK activity by downregulating cyclins, leading to Rb dephosphorylation and inhibition of E2F activity, affecting G_1_ progression and G_1_/S transition (38). While HDACi-induced growth arrest is mainly linked to p21 induction, some evidence points to a p21-independent pathway. For instance, Trichostatin A (TSA) activates the p15^Ink4b^ gene, inducing growth inhibition in colon cancer cells lacking p21 (39). Additionally, a study by Yamashita et al. (40) showed that TSA increased acetylation of histones H3 and H4 in hepatocellular carcinoma cells, causing G_0_/G_1_ phase arrest.

#### 4.1.2 G2/M cell cycle transition

In addition to regulating the G_1_/S transition, HDACi have also been shown to interfere with the G_2_/M transition. HDAC1 knockdown in tumor cells disrupts G_2_/M progression and inhibits cell growth (41). Li et al. (42) demonstrated that HDAC10 controls G_2_/M transition by regulating cyclin A2 expression. Similarly, Kim et al. (43) showed that MHY218, a hydroxamic acid derivative, induces G_2_/M arrest in colon cancer cells through p21 upregulation, independent of p53. TSA further supports this by increasing p21 levels while decreasing Cyclin B1, Plk1, and Survivin, delaying G_2_/M progression (44). Interestingly, suberoylanilide hydroxamic acid (SAHA) causes a G_1_ phase block at low concentrations, but both G_1_ and G_2_/M blocks at higher concentrations (45).

#### 4.1.3 Other ways

Beyond transcriptional suppression of cell cycle genes at G_1_/S and G_2_/M checkpoints, HDACs may also regulate the cell cycle through transcription-independent mechanisms. For instance, the HDAC3-AKAP95/HA95 Aurora B pathway is essential for normal mitosis, as shown by Li et al (46). Additionally, LBH589, an HDAC inhibitor, prevents the degradation of Aurora A and B kinases by inhibiting HDAC3 and HDAC6, leading to G_2_/M arrest and apoptosis in renal cancer cells (47). HDAC3 also regulates the stability of cyclin A via acetylation, which is crucial for S phase progression and mitosis entry (48).

Overall, HDACi can block the cell cycle at the G_1_/S or G_2_/M phases, highlighting HDAC as a potential therapeutic target for abnormal cell growth in cancer.

### 4.2 Apoptosis

The regulation of apoptosis—a genetically programmed cell death mechanism—is intricately governed by the dynamic interplay between pro-survival and pro-apoptotic signals, with HDACs emerging as pivotal modulators of this life-or-death equilibrium. HDACs exert dualistic control over apoptotic pathways through both epigenetic silencing of apoptosis-related genes and direct post-translational modification of key apoptotic executors. By deacetylating histones (e.g., H3K9, H4K16) at promoters of pro-apoptotic factors (*BAX*, *PUMA*, *NOXA*) and tumor suppressors (*p53*), HDACs enforce transcriptional repression under homeostatic conditions, favoring cell survival. Conversely, stress stimuli (e.g., DNA damage, oxidative stress) trigger HDAC inhibition or degradation, leading to chromatin relaxation and reactivation of apoptotic machinery. Apoptosis has been shown to be induced through two major signaling pathways, referred to as the endogenous and exogenous pathways, mediated by mitochondria and death receptors, respectively (49). This section Outlines the relationship between HDAC and apoptosis in terms of different apoptotic pathways, dissects their significance in the development and treatment of cancer, and evaluates strategies for selectively manipulating HDAC activity to restore apoptosis.

#### 4.2.1 Extrinsic pathway

The extrinsic apoptotic pathway, initiated by extracellular death ligands (e.g., FasL, TRAIL) binding to transmembrane receptors (e.g., Fas, DR4/DR5), is tightly regulated by HDACs through both epigenetic and non-epigenetic mechanisms. HDACs modulate this pathway by altering the expression of death receptors, suppressing pro-apoptotic signaling, or directly modifying key apoptotic executors, thereby influencing cellular sensitivity to extrinsic death stimuli. The regulation of HDACi on TRAIL-induced apoptosis has been described in detail in the review of Fulda et al. (50), including the up-regulation of TRAIL receptor expression on the cell surface and the down-regulation of anti-apoptotic proteins (such as cFLIP) by HDACi, so that cancer cells are in a state of TRAIL-triggered apoptosis. Xia et al. (51) confirmed that downregulation of *c-Jun* expression in response to osmotic pressure was caused by transcriptional repression through caspase-7-dependent HDAC3 cleavage, which involved FAS ligand and MEK2-dependent caspase-8 activation. The downregulation of c-Jun promoted the osmotic stress-induced apoptosis.

#### 4.2.2 Intrinsic pathway

The intrinsic (mitochondrial) apoptotic pathway, activated by intracellular stress signals such as DNA damage, oxidative stress, or metabolic imbalance, is critically regulated by HDACs through their dual roles in chromatin remodeling and direct modulation of mitochondrial apoptosis executors. HDACs orchestrate the balance between pro-survival and pro-apoptotic Bcl-2 family proteins, govern mitochondrial outer membrane permeabilization (MOMP), and fine-tune caspase activation, thereby determining cellular commitment to life or death. Activation of the intrinsic apoptotic pathway appears to be the major mechanism by which HDACi induces tumor cell death. In many cases, HDACi activates the intrinsic pathway by up-regulating some BH3-only pro-apoptotic Bcl-2 family genes including *Bim*, *Bid* and *Bmf (* 52). Furthermore, recent studies have shown that HDACi can comprehensively alter the expression of pro-survival and pro-apoptotic Bcl-2 family genes, suggesting a pro-apoptotic biological response (53). Emerging evidence highlights the tumor-suppressive effects of HDAC2 depletion across cancer types. Jung et al. (54) demonstrated that HDAC2 silencing inhibits proliferation and induces apoptosis in human lung cancer cells via coordinated activation of p53 and Bax alongside Bcl-2 downregulation. Notably, Kim et al. (55) further revealed that targeted HDAC2 inactivation in gastric cancer cells restores pro-apoptotic activity of Bax, AIF, and Apaf-1 while suppressing Bcl-2, independent of p53 protein level alterations. This suggests HDAC2 ablation triggers caspase-dependent apoptosis through p53-independent mechanisms. However, the role of p53 in HDACi-mediated apoptosis remains context-dependent (56–59). Sonnemann et al. (60) observed that vorinostat, apicidin, and valproic acid exert antitumor effects largely independent of p53 status, whereas entinostat-induced cytotoxicity partially relies on p53 functionality. These findings collectively underscore the heterogeneous p53 dependency among HDACis, emphasizing the need for compound-specific mechanistic characterization. Notably, MHY218, an HDACi, significantly increased the Bax/Bcl-2 ratio in a concentration-dependent manner and activated caspases-3, -8, and -9, indicating that it induces apoptosis in colon cancer cells via both intrinsic and extrinsic pathways (43). It seems that the different effects of HDACi in the same cell type may be caused by the structural characteristics of the different HDACi.

### 4.3 DNA damage and DNA repair

The maintenance of genomic stability relies on a sophisticated network of DNA damage response (DDR) pathways that detect, signal, and repair lesions to prevent mutagenesis and cell death. HDACs, traditionally recognized as chromatin-modifying enzymes, have recently been implicated as critical regulators of DDR through their dual roles in modulating chromatin architecture and direct interactions with DNA repair machinery. DNA double strand breaks (DSBs) are lethal lesions detected by DNA damage signaling mechanisms, and most are repaired by non-homologous end-joining (NHEJ) or homologous recombination (HR) (61). NHEJ is a highly efficient process that joins DNA ends with minimal processing (62), while HR uses undamaged homologous sequences to ensure precise repair (63). Miller et al. (64) demonstrated that loss of HDAC1 or HDAC2 renders cells highly sensitive to DNA-damaging agents and exhibit persistent DNA damage signals, phenotypes that confirm defective DSBs repair, particularly through the NHEJ pathway. Specifically, depletion of HDAC9 or HDAC10 by RNA interference can specifically inhibit HR, leading to increased sensitivity to mitomycin C (65). Ataxia-telangiectasia mutated (ATM) is a master regulator of DNA damage response, promoting the activation of *BRCA1*, *CHK2*, and *p53*, which induces DNA repair response genes such as p21, *GADD45A*, and *RRM2B*. Previous studies by Thurn et al. (66) confirmed that selective silencing of HDAC1 and HDAC2 *in vitro* and *in vivo* is sufficient to modulate ATM activation, reduce *GADD45A* and *RRM2B* induction, and increase sensitivity to DNA strand breaks. Moreover, another study (67) found that silencing *HDAC2 gene* could alleviate the ATM/p53-mediated cell death pathway in osteosarcoma U2OS cells in response to adriamycin, confirming that HDAC2 is involved in the early molecular events of DNA damage response and is a coactivator of p53. HDAC6 has long been thought to play a unique role due to its cytosolic localization and ability to deacetylate non-histone proteins (68). The study by Yang et al. (69) confirmed that HDAC6 can regulate DDR-related genes by affecting Sp1 expression, eliminating DNA damage induced by MPT0B291, an HDAC6 inhibitor, providing evidence for MPT0B291 as a potential compound for glioblastoma (GBM) therapy. Strikingly, recent studies have revealed that HDAC6 functions as a valine sensor, which is retained in the nucleus upon valine deprivation, where it binds to and deacetylates ten-eleven translocation 2 (TET2). This process promotes DNA damage through thymine DNA glycosylase (TDG)-driven excision, thereby unveiling a novel therapeutic strategy for cancer treatment (70). In particular, in the latest study by Yao et al. (71), IHCH9033, a new selective class I HDACi, can selectively inhibit DNA repair in FLT3-ITD AML cells, leading to DNA damage accumulation and overcoming resistance to FLT3 inhibitors. Research on HDACi in DNA damage and repair is still developing, but it is known that therapeutic doses of HDACi not only induce DNA damage but also impair repair mechanisms, sensitizing cells to ionizing radiation (IR), topoisomerase inhibitors, and cisplatin (72–74).

Mammalian sirtuins (SIRT 1-7), which belong to NAD+ -dependent class III HDACs, have different modes of action, targets and subcellular compartments, and play unique roles in DNA damage and repair. SIRT1, a NAD^+^-dependent deacetylase, has been demonstrated to directly interact with and deacetylate multiple core DNA repair factors, including Ku70 (15) (critical for non-homologous end joining), apurinic/apyrimidinic endonuclease-1 (APE1) (base excision repair effector) (75), NBS1 (nibrin/p95, a central component of the MRE11-RAD50-NBS1 [MRN] complex for DNA damage sensing) (76), and xeroderma pigmentosum group A (XPA) (key mediator of nucleotide excision repair) (77). This post-translational modification modulates their enzymatic activities, subcellular localization, and recruitment to DNA lesion sites, thereby fine-tuning repair fidelity across distinct DNA damage response pathways. The relationship between SIRT1 and DNA damage response has been elaborated in recent reviews, including the interaction with different proteins in the major DNA repair mechanisms and DDR pathway, recruiting them to DNA damage foci or activating proteins involved in DNA repair through deacetylation (78). Previous studies have found that SIRT5 is highly expressed in colorectal cancer (CRC), and knockdown of SIRT5 impairs the production of ribo-5-phosphate, which is required for nucleotide synthesis, resulting in continuous and irreparable DNA damage. This results in CRC cell cycle arrest and significant apoptosis, indicating that SIRT5 can be a promising target for CRC therapy (79). SIRT6 plays a critical role in DNA repair by enhancing resistance to DNA damage and maintaining genomic stability, particularly through base excision repair (BER), as demonstrated by Mostoslavsky et al. (80). Moreover, SIRT6-deficient mice exhibit decreased chromatin-associated levels of SNF2H in specific tissues, a finding that correlates with elevated DNA damage (81). In addition, research by Mao et al. (82) has shown that SIRT6 interacts with poly(ADP-ribose) polymerase 1 (PARP1) and catalyzes its mono-ADP-ribosylation at lysine 521, thereby enhancing PARP1 activity and facilitating the repair of DSBs under oxidative stress conditions. Members of the SIRT family play multifaceted roles in DNA damage repair, exerting effects that are equally as important as those of classical HDACs.

### 4.4 Autophagy

The interplay between HDACs and autophagy represents a pivotal nexus in cellular adaptation to metabolic stress, genomic instability, and environmental challenges. Emerging evidence underscores HDACs as dual regulators of autophagy, operating through both epigenetic and non-epigenetic mechanisms to fine-tune autophagic flux. By deacetylating histones (e.g., H3K9, H4K16) at promoters of autophagy-related genes (*ATG5*, *LC3*, *BECN1*), HDACs suppress basal autophagy under nutrient-replete conditions, while their inhibition or stress-induced degradation triggers transcriptional activation of autophagic machinery (83–85). The effects of autophagy on cancer development and the interaction between autophagy and HDAC have been described in detail in the latest review by Koeneke et al (86).

Early work by Moresi et al. (87) demonstrated that the simultaneous deletion of HDAC1 and HDAC2 in mice inhibited autophagosome formation. Similarly, Kang et al. (88) found that knockdown of HDAC4 induced autophagy, increasing LC3-II, Beclin-1, and ATG7 levels. Not only that, the researchers found that LC3-II increased over time when HDAC5 was depleted in breast cancer cells, and this effect was enhanced with the use of lysosomal inhibitors, suggesting that HDAC5 downregulation increased autophagic flux (89). HDAC6 is involved in the regulation of autophagy at multiple levels, including post-translational modification (PTM) of transcription factors involved in autophagy-related transcription (90, 91), formation of aggregates routinely cleared through the autophagic pathway (92, 93), and transport and degradation of autophagosomes (94). Liu et al. (95) observed that the level of acetylated phosphotidylethanolamine (PE)-conjugated LC3B (LC3B-II) was increased in cells treated with tubacin, a specific inhibitor of HDAC6, under normal medium. However, tubacin only partially inhibited serum starvation-induced deacetylation of LC3B-II, suggesting that HDAC6 is not the only deacetylase acting on LC3B-III during serum starvation-induced autophagy. Therefore, HDAC6 depletion impairs serum starvation-induced autophagy. The post-transcriptional modification (PTM) of autophagy-related transcription factors, such as transcription factor EB (TFEB) and forkhead box 1 (FOXO1), notably affects their activity and thus regulates the autophagy-lysosome pathway (96), and current studies have reported that HDAC6 deacetylates TFEB and FOXO1 to reduce their activity and inhibit autophagy (91, 97, 98). In addition, in a study (99) on breast cancer, it was also found that HDAC6 knockdown resulted in reduced LC3B protein and reduced autophagy. Another work (100) showed that productive autophagy with efficient autophagosome-lysosome fusion is dependent on HDAC10 and that depletion of HDAC10 enhances the sensitivity of tumor cells to chemotherapeutic agents such as doxorubicin.

Sirtuins on autophagy in cancer has been detailed in a recent review by Aventaggiato et al. (101) and will not be repeated here.

### 4.5 Angiogenesis

Hypoxia is a common characteristic of many solid tumors, where tumor cells in hypoxic regions adapt to low oxygen conditions by activating several survival pathways. Among these, the activation of the hypoxia-inducible factor-1 (HIF-1) transcription factor is the most well-known mechanism employed by hypoxic cells in this hostile microenvironment. Notably, there is a strong correlation between HIF-1 and tumor angiogenesis (102). As a result, drugs that inhibit HIF-1 expression hold significant potential as antitumor agents. HIF-1 is a heterodimeric transcription factor, consisting of two subunits: HIF-1α (or its analogs HIF-2α and HIF-3α) and HIF-1β. Histone deacetylase inhibitors (HDACIs) have been shown to significantly reduce HIF-1α expression and are currently being evaluated in clinical trials, partly due to their potent anti-angiogenic effects. However, the precise mechanism by which HDACIs function as HIF-1α inhibitors remains unclear.

The research of Yoo et al. (103) demonstrated that the expression of metastasis-associated protein 1 (MTA1) is strongly induced under hypoxic conditions, and MTA1 promotes the deacetylation of HIF-1α by upregulating histone deacetylase 1 (HDAC1). In a separate study by Geng et al. (104), it was shown that acetylation of the HIF-1α protein increased with HDAC4 shRNA and decreased with HDAC4 overexpression. In contrast, HDAC5 and 6 promote the maturation and stabilization of HIF-1α by deacetylating its chaperones HSP70 and HSP90 (105, 106). Lim et al. (107) observed that SIRT1-mediated deacetylation of HIF-1α at Lys674 inhibits HIF-1α activity by preventing the recruitment of p300. Under hypoxic conditions, SIRT1 inhibition creates a positive feedback loop that maintains high levels of HIF-1 activity.

Vascular endothelial growth factor (VEGF) is a key angiogenic factor that promotes angiogenesis in various pathological conditions, including inflammation, ischemic diseases, and cancer. In a report by Ray et al. (108) involving breast cancer, it was found that Kruppel-like factor-4 (KLF-4) recruits HDAC2 and HDAC3 at the VEGF promoter and represses their transcription, and that upregulation of VEGF in cancer is associated with loss of KLF-4-HDAC-mediated transcriptional repression. In another research (109), it was confirmed that silencing of HDAC5 increased the expression of fibroblast growth factor 2 (FGF2) and angiogenesis directing factors including Slit2. Kaluza et al. (110) found that HDAC6 interacts with and deacetylates the actin remodeling protein cortactin in endothelial cells (ECs), thereby regulating endothelial cell migration and germination. However, in another study, it was found that knockdown of HDAC6 significantly upregulated the expression of HIF-1α and VEGFA *in vivo* and *in vitro* and promoted HIF-1α-mediated hepatocellular carcinoma (HCC) angiogenesis (111). Turtoi et al. (112) were the first to show that HDAC7 epigenetically targets the AKAP12 tumor/angiogenesis suppressor gene. Not surprisingly, HDAC regulates angiogenesis through a variety of pro- and anti-angiogenic factors.

### 4.6 Metastasis

Epithelial-mesenchymal transition (EMT) is a critical process in cancer cell invasion and metastasis, with recent studies highlighting the role of histone deacetylases (HDACs) in regulating EMT across various cancers. EMT is marked by the loss of the epithelial cell marker E-cadherin (CDH1), and several transcriptional repressors of CDH1 have been identified, including Snail, Slug, Twist, ZEB1, and ZEB2. The breakdown of the basement membrane (BM) barrier allows cancer cells to directly invade the surrounding stromal region, a process driven by active proteolysis, primarily through the action of matrix metalloproteinases (MMPs) (113).

The recruitment of HDACs to the CDH1 promoter has been shown to be regulated by the transcription factor ZEB1, as first demonstrated by Aghdassi et al. in their study on pancreatic cancer (114). In research on pancreatic cancer (115), the genetic inactivation of E-cadherin (encoded by the CDH1 gene) was found to induce EMT and promote metastasis *in vivo*. The silencing of E-cadherin was mediated by a transcriptional repressor complex involving Snail, HDAC1, and HDAC2. Additionally, this Snail/HDAC1/HDAC2 complex is essential for EZH2-mediated repression of CDH1 in nasopharyngeal carcinoma cells (116). A previous study (117) confirmed that class I HDAC inhibitors enhance the acetylation of Y-box binding protein 1 (YB-1) and increase oxidative stress, thereby blocking sarcoma metastasis. More recently, it was shown that DNTTIP1 represses DUSP2 gene expression by recruiting HDAC1 to its promoter, maintaining the deacetylated state of histone H3K27 (118), and downregulation of DUSP2 leads to abnormal activation of ERK signaling and elevated MMP2 levels, which promote metastasis in nasopharyngeal carcinoma (NPC). In a research by Ma et al. (119), the cytoplasmic expression of HDAC3 was found to be upregulated in brain metastases from breast cancer, while its nuclear expression was conversely downregulated. This suggests that HDAC3 plays a key role in the development and progression of brain metastases in breast cancer patients, though the study did not explore the underlying mechanisms in detail. Additionally, heat shock protein 90 (HSP90), another non-histone substrate of HDAC6, is primarily responsible for promoting protein maturation and maintaining protein structure (120), which is crucial for the stability and function of proteins involved in tumor metastasis (121). Recent studies (122) have shown that targeted inhibition of HDAC6 increases the acetylation of HSP90, which weakens the binding between HSP90 and ATP, thereby reducing the interaction between chaperone proteins and oncogenes. Additionally, cytoplasmic linker protein 170 (CLIP-170), a microtubule-binding protein, regulates cell motility by modulating microtubule dynamics. In a study by Li et al. (123) HDAC6 was found to interact with CLIP-170 and the two proteins acted together to stimulate the migration of pancreatic cancer cells. Additionally, HDAC6, a novel estrogen-regulated gene, was found to have increased expression in estrogen receptor-positive breast cancer MCF-7 cells, as reported by Saji et al (124). Elevated HDAC6 expression enhanced cell motility by promoting its binding to α-tubulin and increasing microtubule (MT) activity. This finding was further confirmed in a separate study on neuroblastoma (125). HDAC8 is highly expressed in breast cancer compared to other types of cancers. In a study (126) triple-negative breast cancer (TNBC), HDAC8 was found to promote TNBC cell migration by regulating Hippo-YAP signaling. Additionally, HDAC8 was shown to drive breast cancer cell dissemination through the AKT/GSK-3β/Snail signaling pathway (127). Not only that, HDAC8 cooperates with the SMAD3/4 complex to inhibit *SIRT7* and promote cell survival and migration (128). HDAC8 promotes cancer metastasis by suppressing maspin expression in prostate cancer (129). HDAC8 also promotes glioma migration by regulating the acetylation levels of α-tubulin (130). Additionally, the long non-coding RNA (lncRNA) ID2-AS1, which downregulates inhibitor of DNA binding 2 (ID2), enhanced ID2 transcription by blocking HDAC8 occupancy at the ID2 enhancer region, thereby inhibiting hepatocellular carcinoma (HCC) invasion and migration both *in vitro* and *in vivo* (131). In a study by Song et al. (132) on cervical cancer, it was confirmed that HDAC10 suppresses the expression of MMP2 and MMP9, genes known to be critical for cancer cell invasion and metastasis. Furthermore, HDAC11 was found to inhibit the migration and invasion of colorectal cancer cells by downregulating MMP3 expression (133).

In prostate cancer, SIRT1 was found to promote cell migration *in vitro* and metastasis *in vivo* by synergistically inhibiting CDH1 transcription alongside ZEB1 (134). Furthermore, a study by Eades et al. (135) on breast cancer showed that SIRT1 overexpression was associated with reduced levels of miR-200a, which normally acts as a negative regulator of SIRT1 and suppresses EMT. A similar mechanism was identified in oral cancer, where research by Chen et al. (136) demonstrated the key role of the SIRT1/Smad4/MMP7 pathway in the EMT process.

### 4.7 Immunity

HDACs, beyond their canonical roles in chromatin remodeling and transcriptional repression, have emerged as pivotal regulators of immune cell differentiation, activation, and functional polarization. By dynamically modulating the acetylation status of both histones (e.g., H3K9, H4K16) and non-histone immune-related proteins (e.g., STATs, NF-κB, Foxp3), HDACs fine-tune the expression of cytokines, chemokines, and immune checkpoint molecules, thereby shaping innate and adaptive immune responses. Early studies by Xiao et al. (137) have confirmed that HDAC5 can reduce immune responses and the *de novo* expansion of T regulatory (Treg) cells, highlighting the importance of HDAC5 in antitumor immune responses. Recent research has also found that HDAC5 regulates PD-L1 expression by directly interacting with NF-κB p65. Therefore, silencing or inhibiting HDAC5 in pancreatic ductal adenocarcinoma can sensitize tumors to immune checkpoint blockade therapy (138). Foxp3 is a key transcription factor of Treg cells, and its expression and activity level are determined by post-translational modification. In the transplantation environment, regulating the acetylation or deacetylation of key lysine residues of Foxp3 can promote its stability and function, thus regulating the generation and activity of Tregs. The role of HDACs in regulating Treg function has been described in detail in the latest review by Wang et al (139). In particular, the latest research found that targeting HDAC3 could enhance CXCL12 secretion through the ATF3 dependent pathway, thereby stimulating the recruitment and activation of NK cells and inhibiting the progression of T-cell lymphoma (140). Regarding HDACi, ACY241, a selective inhibitor of HDAC6, has been shown to significantly reduce the frequency of CD138^+^MM cells, CD4^+^CD25^+^FoxP3^+^ regulatory T cells, and HLA-DR^Low^/-CD11b^+^CD33^+^ myeloid-derived suppressor cells. Reduced immune checkpoint PD1/PD-L1 expression in CD8^+^T cells and bone marrow cells from myeloma patients. ACY241 increases the expression of B7 (CD80, CD86) and MHC (class I, class II) in tumor and dendritic cells. ACY241 also enhanced the antitumor activity of antigen-specific cytotoxic T lymphocytes (CTL) in a dose - and time-dependent manner, as indicated by increased production of perforin/CD107a, IFN-γ/IL-2/TNF-α, and antigen-specific central memory CD8+ T cells (141).

### 4.8 Stem cells

Stem cells, with their unparalleled capacity for self-renewal and lineage-specific differentiation, serve as the cornerstone of tissue homeostasis and regenerative medicine. HDACs, long recognized as epigenetic modifiers, have recently emerged as master regulators of stem cell fate, dynamically balancing pluripotency maintenance and differentiation through both chromatin-dependent and -independent mechanisms. In the latest study (142), inhibition of histone deacetylase activity in LSD1-HDAC1/2 corepressor complex by sodium butyrate (NaB) increased the number of 2C-like cells in mouse embryonic hepatocytes and directly reprogrammed embryonic hepatocytes into trophoblast stem cells. Paradoxically, dysregulated HDAC activity drives pathological stemness in cancer stem cells (CSCs), where aberrant deacetylation stabilizes oncogenic transcription programs (e.g., Wnt/β-catenin, Notch) and confers therapy resistance (143). This duality underscores the context-dependent roles of HDAC isoforms, positioning them as both guardians of regenerative capacity and accomplices in malignant transformation. The latest study (144) found that the combination of HDACi CS055 and chiglitazar can synergistically act on leukemia cell lines and leukemia stem cell-like cells in patient samples. Chiglitazar enhanced the inhibitory effect of CS055 on HDAC3 by down-regulating the expression of SLC7A11, an inhibitor of ferroptosis, and induced ferroptosis in leukemia stem cell-like cells. HDAC profoundly affects the pluripotency, differentiation and therapeutic application of stem cells by regulating chromatin dynamics and signaling pathways, which still needs to be further explored in the future.

## 5 Conclusion

HDAC and HDACi have shown extraordinary potential in the treatment of cancer, and widely regulate the key processes of tumorigenesis. However, they still face many challenges. Firstly, the efficacy of HDACi as a single agent in solid tumors is insufficient, which may be partially related to tumor heterogeneity, microenvironment hypoxia and poor drug permeability (145, 146). Moreover, most of the existing HDACis are broad-spectrum inhibitors, lack of subtype selectivity, and have hematologic and cardiac-related toxicity (147). In addition, predictive markers for efficacy of HDACi have not been clearly defined, leading to a dilemma in patient stratification. In summary, HDAC and HDACi are the new candidates for epigenetic regulation, and their future development needs to break through the bottleneck of solid tumor efficacy, improve subtype selectivity, and further identify HDAC-related markers to guide clinical diagnosis and treatment. Combined treatment strategies (such as HDACi + immunotherapy or targeted drugs) and precision medicine models (individualized treatment based on molecular subtypes) are the directions we need to actively explore in the future to finally achieve the “precision endurance” of cancer treatment.
